# Novel use of intravascular lithotripsy for vertebral artery stenosis: A case report

**DOI:** 10.1016/j.radcr.2025.06.018

**Published:** 2025-06-27

**Authors:** Orlando Diaz, Jochen Gerstner-Saucedo, Isabel Carmona

**Affiliations:** a Department of Radiology, Houston Methodist Hospital, Houston, TX, USA; b Advanced Imaging Lab, Department of Radiology, University of Colorado Anschutz Medical Campus, Aurora, CO, USA; c School of Medicine, Faculty of Health Sciences, Universidad Icesi, Cali, Colombia

**Keywords:** Intravascular lithotripsy (IVL), Vertebral artery stenosis, Calcified plaque, Endovascular procedure, Balloon angioplasty, Stent deployment

## Abstract

Severe vertebral artery stenosis complicated by dense calcification represents a significant surgical challenge, particularly when accompanied by limited collateral circulation. Traditional high-pressure balloon angioplasty is an adequate option. However, it can increase the risk of vascular injury and it often achieves inadequate stent expansion. Intravascular lithotripsy (IVL) has recently been proposed as an alternative method for safely fracturing calcified plaques with acoustic pressure waves at lower balloon pressures. We report a symptomatic patient where IVL was used for predilation during a vertebral artery stenting from a femoral approach. Initially, diagnostic imaging revealed a hypoplastic left vertebral artery terminating in the posterior inferior cerebellar artery (PICA), as well as critical stenosis caused by heavily calcified plaque in the V2 segment of the right vertebral artery. The patient underwent further postdilation and stent deployment following a successful endovascular procedure with IVL at an inflation pressure of 2 atm. The patient experienced complete symptom resolution and recovered without any complications. This case highlights the potential benefits of IVL over traditional angioplasty techniques by demonstrating its efficacy and safety in treating heavily calcified vertebral artery stenosis.

## Introduction

Vertebral artery stenosis is a recognized cause of posterior circulation ischemia and, when left untreated, may lead to disabling neurological events if left untreated. The risk is elevated when collateral flow is limited such as when the contralateral left vertebral artery is hypoplastic or terminates in the posterior inferior cerebellar artery (PICA) [1,2]. In such scenarios, revascularization becomes critical, if the plaque is soft regular angioplasty is recommended, but in sometimes but is often complicated by heavy eccentric calcification, which limits vessel compliance and increases the risk of arterial injury during conventional high-pressure angioplasty [3].

Intravascular lithotripsy (IVL) employs acoustic pressure waves delivered via a balloon-mounted emitter to fracture calcified plaques while preserving vessel integrity. Initially developed for peripheral and coronary interventions, IVL has recently been explored in the treatment of supra-aortic lesions, including those of the carotid [4] and subclavian arteries [5]. To date, however, its application in vertebral artery stenosis has not been reported.

**Fig. 1:**
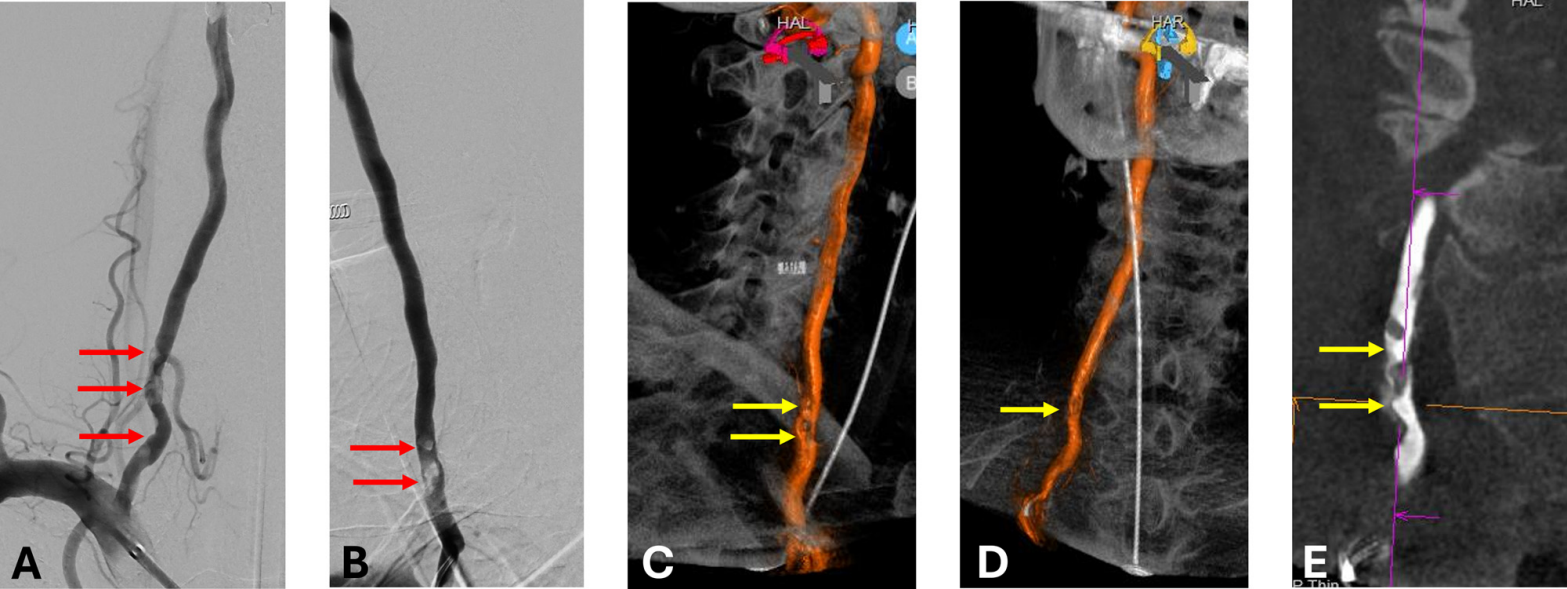
Right vertebral artery angiography and cross-sectional imaging. (A) Anteroposterior digital subtraction angiography (DSA) and (B) lateral DSA demonstrate a critical stenosis of the V2 segment (red arrows). (C–D) Volume-rendered 3D reconstructions and (E) DynaCT show a heavily calcified, eccentric plaque causing luminal narrowing at the same level (yellow arrows).

**Fig. 2:**
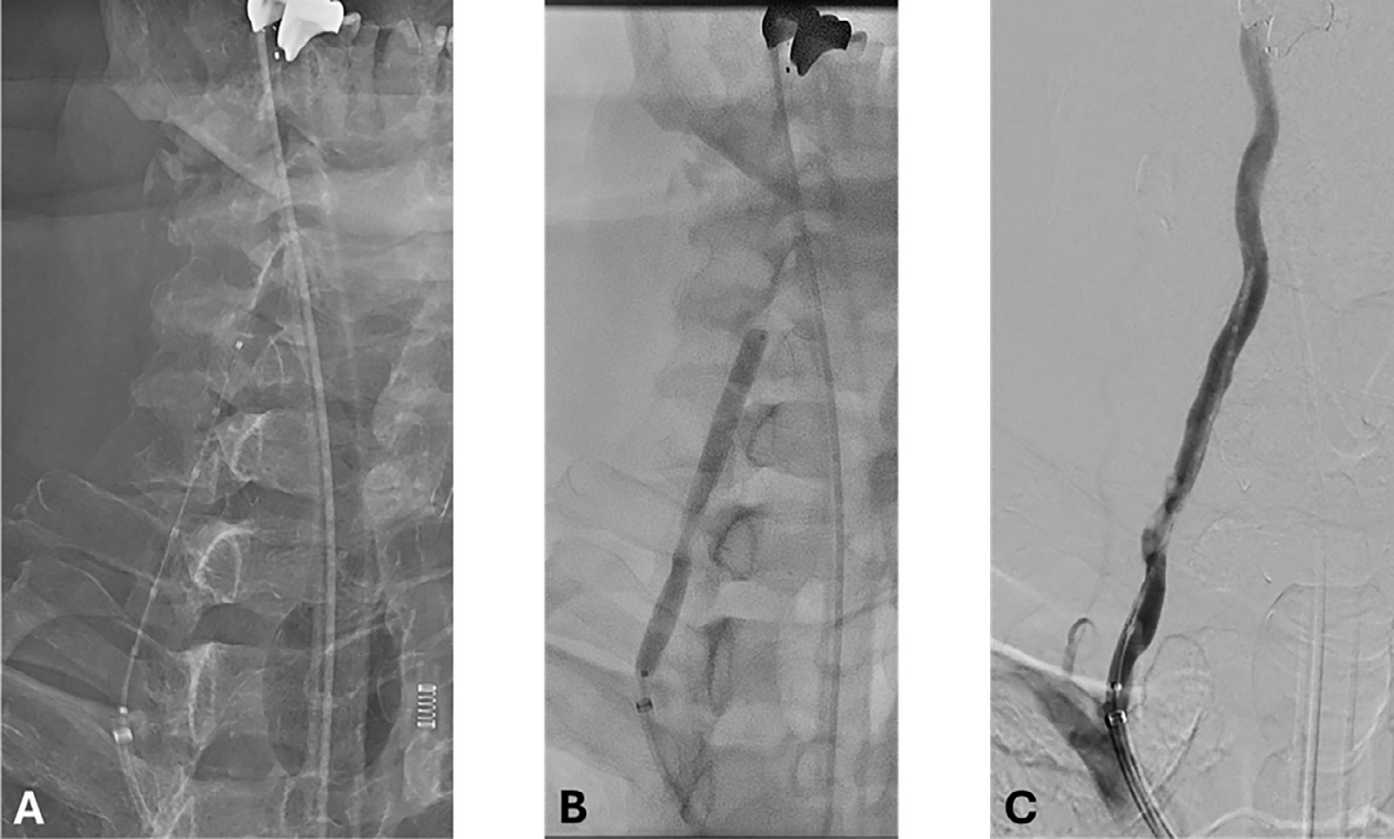
Intraprocedural fluoroscopy and digital subtraction angiography during vertebral artery intervention. A–B: Fluoroscopic images demonstrating inflation of a 5 mm × 60 mm Shockwave intravascular lithotripsy (IVL) balloon (Shockwave Medical) across the calcified V2 segment of the right vertebral artery. C: Digital subtraction angiography following IVL shows improved blood flow through the treated arterial segment.

**Fig. 3:**
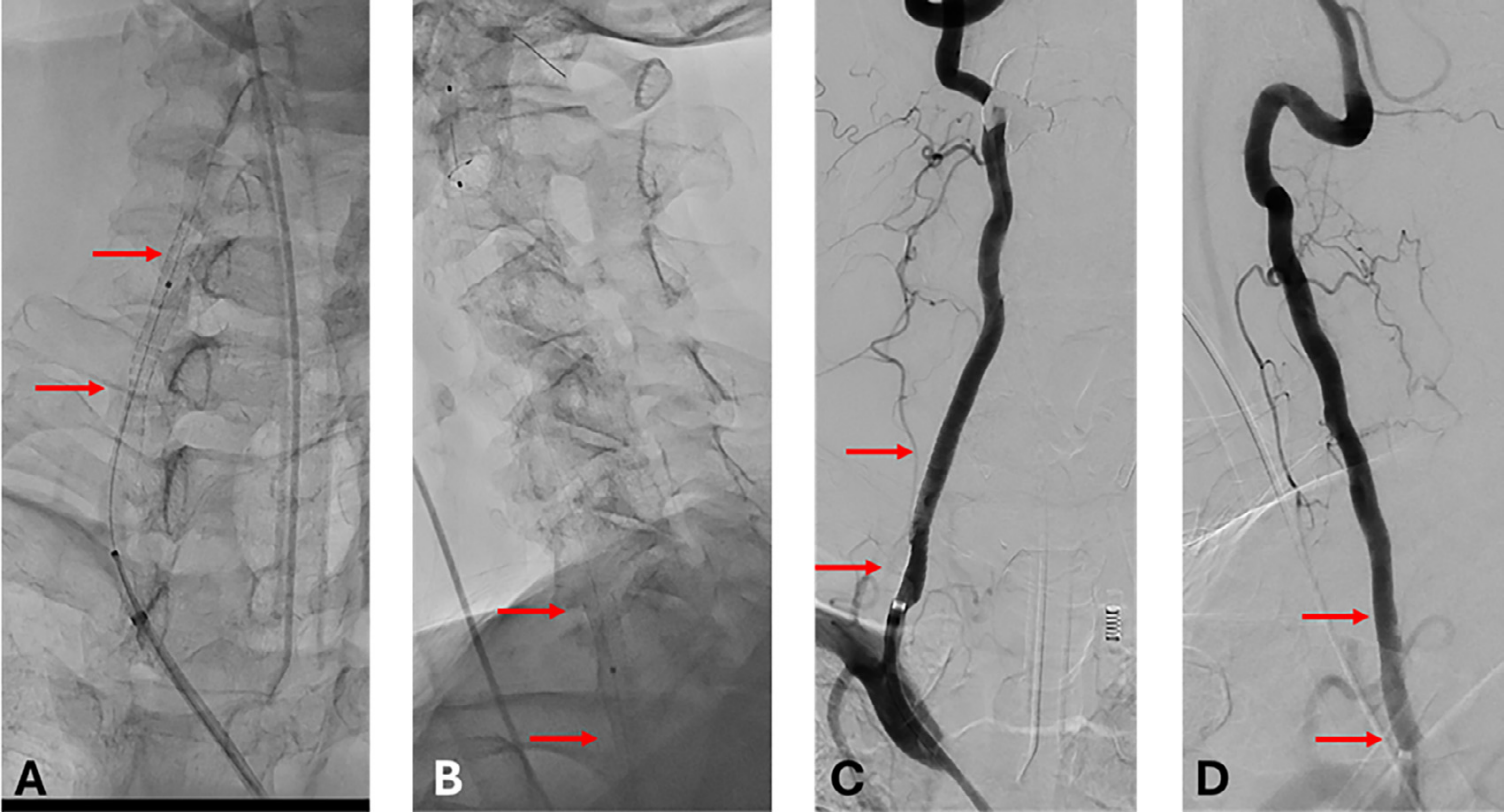
Stent deployment and postprocedural imaging. (A, B) Anteroposterior and lateral fluoroscopic views demonstrate the deployment and positioning of a 5 × 40 mm stent (red arrows) across the V2 segment of the right vertebral artery. (C, D) Anteroposterior and lateral DSA projections showing poststenting luminal restoration and brisk flow without residual stenosis (red arrows).

In this case report, we describe what we believe is the first reported use of IVL to treat a heavily eccentric calcified stenosis of the vertebral artery in an elderly patient with vertebrobasilar insufficiency, limited collateral flow, and high procedural risk. This case highlights the procedural considerations, technical success, and immediate safety of IVL in the posterior circulation Fig. 1, Fig. 2, Fig. 3.

## Case presentation

An 82-year-old male with a history of hypertension, hyperlipidemia, hepatocellular carcinoma, prostate cancer, prostatic hyperplasia, and tobacco use, presented with a 6-month history of mild vertigo, intermittent diplopia, visual abnormalities, and postural instability. His current treatment consisted of dual antiplatelet therapy (clopidogrel and aspirin) and atorvastatin.

Neurological examination was unremarkable. Vital signs were stable (BP: 142/70 mmHg; HR: 71 bpm; SpO₂: 98%). Cerebral angiography demonstrated a critical 80% stenosis of the V2 segment of the right vertebral artery caused by a heavily calcified eccentric plaque. The left vertebral artery was hypoplastic and terminated in the posterior inferior cerebellar artery (PICA), offering no contribution to the basilar system. Mild stenosis (25%) of the right internal carotid artery was also noted.

The procedure was performed under general anesthesia via right femoral access. A 90 cm BMX 96 sheath (Penumbra, Alameda, CA) was advanced into the right subclavian artery. The lesion was crossed using a 0.014″ wire (Asahi Intecc, Aichi, Japan). A 5 mm SpiderFX embolic protection device (Medtronic, Minneapolis, MN) was deployed in the distal V2 segment. A 5 mm × 60 mm Shockwave IVL balloon (Shockwave Medical, Santa Clara, CA) was inflated to 2 atm, and 3 cycles of 30 pulses (90 pulses total) were delivered. The poststenting angiogram demonstrated recanalization with improved lumen. A 5 mm × 40 mm Precise stent (Cordis, Hialeah, FL) was deployed from distal V1 to distal V2, followed by postdilation with a 5 mm × 20 mm noncompliant balloon (Sterling, Boston Scientific, Marlborough, MA). Final angiography demonstrated full stent expansion and widely patent artery. No debris was noted upon retrieval of the protection device. Post treatment cerebral arteriogram showed no embolic events were.

The patient was monitored in the intensive care unit postprocedure and remained neurologically intact. No perioperative complications occurred. At the time of follow-up, the patient remains asymptomatic. Repeat imaging is scheduled at 6 months.

## Discussion

IVL was successfully employed in this case to treat a heavily calcified vertebral artery stenosis in a patient with vertebrobasilar insufficiency and limited collateral flow. To our knowledge, this represents the first reported application of IVL in the vertebral circulation.

The endovascular treatment of heavily calcified arterial lesions remains a technical challenge, particularly in vessels with poor compliance and increased rigidity created by calcified plaques. In these settings, traditional angioplasty may require high-pressure inflation (up to 18 atm) which further elevates the risk of arterial trauma [3].

IVL provides and alternative by delivering acoustic pressure waves via a low-pressure balloon, selectively fracturing the calcium layers in the plaque while preserving the vessel wall [3,6] The safety of IVL has been demonstrated in preclinical trials, which show no histologic damage even at high energy levels [6].

IVL has shown favorable outcomes in the treatment of peripheral and coronary artery disease, with trials like Disrupt PAD III reporting higher patency rates in femoropopliteal lesions at 1- and 2-year follow-up [3,7]. More recently, It has also been applied in challenging supra-aortic territories, including the subclavian and carotid arteries [4,5,8]. The technical challenges of these procedures are similar to those of the vertebral circulation, such as the need for embolic protection because of their proximity to the brain, and vessel tortuosity.

IVL has been used successfully used to treat severely calcified carotid artery stenosis, allowing for efficient stent expansion and minimizing mechanical stress [4]. Similar outcomes have been reported in IVL-assisted stenting of calcified subclavian artery lesions, which are anatomically adjacent and procedurally comparable to the vertebral origin due which share access routes and procedural challenges [5]. The IVL catheter can also be used in very tortuous or small-caliber vessels [3].

While IVL offers advantages, limitations persist. Current IVL devices have a restricted size portfolio, with the smallest available balloon at 2.5 mm, limiting applicability in small-caliber or distal vessels. It is also worth noting that, long-term outcome data specific to neurovascular uses is scarce [7,8].

This case supports the use of IVL as a safe and effective alternative when conventional approaches are contraindicated or insufficient. While the experience with vertebral interventions remains limited, we consider that this is the first case in the literature and our findings contribute to the expanding use of IVL in complex cerebrovascular cases. Larger studies are needed to validate these findings and define long-term efficacy.

## Patient consent

Written informed consent was obtained from the patient for publication of this case report and the accompanying images.
